# Exercise modality–dependent non-hypertrophic anabolic adaptations in skeletal muscle of rats with type 2 diabetes

**DOI:** 10.3389/fphys.2026.1773632

**Published:** 2026-03-03

**Authors:** Yi-dan Zhang, Xiang-xing Zhao, Xia Liu

**Affiliations:** 1 School of Physical Education, Hunan Normal University, Changsha, China; 2 Key Laboratory of Physical Fitness and Exercise Rehabilitation of Hunan Province, Hunan Normal University, Changsha, China; 3 Key Laboratory of Adolescent Physical Fitness Research and Monitoring, Hunan Normal University, Changsha, China

**Keywords:** aerobic exercise, irisin, muscle fibrosis, muscle protein turnover, resistance exercise, skeletal muscle atrophy, ubiquitin–proteasome system

## Abstract

**Background:**

Type 2 diabetes mellitus (T2DM) is frequently accompanied by progressive skeletal muscle loss and dysfunction, commonly referred to as diabetic sarcopenia. Exercise is an established non-pharmacological therapy for T2DM; however, how different exercise modalities differentially influence skeletal muscle protein regulation and anabolic signaling remains unclear. This study compared the effects of aerobic exercise, resistance exercise, and their combination on skeletal muscle protein content and irisin-associated anabolic signaling in a rat model of T2DM.

**Methods:**

Male rats with diet- and streptozotocin-induced T2DM were assigned to aerobic exercise, resistance exercise, combined aerobic–resistance exercise, or sedentary diabetic control for 8 weeks. Metabolic indices, skeletal muscle mass and morphology, and molecular markers related to muscle protein content, proteolytic signaling, and anabolic pathways were assessed using biochemical, histological, and protein expression analyses.

**Results:**

Diabetic rats exhibited impaired metabolic profiles, reduced skeletal muscle mass and protein content, and increased muscle fibrosis. All exercise modalities improved selected metabolic indices. Exercise intervention significantly increased skeletal muscle protein content across all exercise groups, despite minimal changes in muscle fiber cross-sectional area and no detectable suppression of canonical ubiquitin–proteasome atrophy markers. Resistance-containing exercise modalities showed greater engagement of integrin α7β1 and PI3K-related signaling components, whereas AKT and mTOR responses were observed across exercise modalities. Exercise increased skeletal muscle irisin expression.

**Conclusion:**

Exercise intervention in diabetic skeletal muscle induces a non-hypertrophic anabolic adaptation characterized by increased muscle protein content and patterned activation of anabolic signaling pathways. Resistance-containing exercise preferentially engaged select mechanotransduction-related signaling components without conferring global superiority across outcomes.

## 1 Introduction

Type 2 diabetes mellitus (T2DM) is a prevalent metabolic disorder characterized by chronic hyperglycemia and insulin resistance, and its global incidence continues to rise with aging populations and sedentary lifestyles. Beyond classical metabolic disturbances, T2DM is increasingly recognized as a systemic disease that profoundly affects skeletal muscle structure and function (Castillo et al., 2025). Skeletal muscle is the largest insulin-sensitive tissue in the body and accounts for the majority of postprandial glucose disposal. Consequently, pathological alterations in skeletal muscle not only represent a complication of diabetes but also contribute directly to the progression of insulin resistance and metabolic dysregulation. A growing body of evidence indicates that individuals with T2DM exhibit accelerated loss of skeletal muscle mass and function, a condition commonly referred to as diabetic sarcopenia, which is associated with impaired mobility, increased frailty, and elevated morbidity and mortality risk (Park et al., 2009; Leenders et al., 2013; Cruz-Jentoft et al., 2018).

The pathophysiology of diabetic sarcopenia is multifactorial and involves disruptions in the balance between muscle protein synthesis and degradation. Chronic hyperglycemia, reduced insulin availability, and impaired insulin signaling can suppress anabolic pathways while promoting catabolic processes within skeletal muscle (Wang et al., 2006). The ubiquitin–proteasome system has been widely implicated in diabetes-associated muscle atrophy, and muscle-specific E3 ubiquitin ligases, including muscle RING finger protein-1 (MuRF1) and atrogin-1, have been reported to be upregulated in certain diabetic contexts, thereby accelerating degradation of myofibrillar proteins (Bodine and Baehr, 2014). In parallel, the phosphatidylinositol 3-kinase (PI3K)/protein kinase B (AKT)/mammalian target of rapamycin (mTOR) signaling pathway, which is essential for insulin-mediated protein synthesis and muscle maintenance (Frost and Lang, 2007; Li et al., 2019), is frequently described as impaired in T2DM. Disruption of this pathway is thought to weaken translational control and exacerbate net protein loss, reinforcing the link between metabolic dysfunction and skeletal muscle deterioration.

Physical exercise is widely recognized as an effective non-pharmacological strategy for improving glucose metabolism and preserving skeletal muscle health in individuals with T2DM. However, distinct exercise modalities induce different physiological and molecular adaptations. Aerobic exercise is well established for its capacity to enhance insulin sensitivity, promote glucose uptake through GLUT4 translocation, and improve mitochondrial function, thereby exerting pronounced metabolic benefits (Richter and Hargreaves, 2013; Sylow et al., 2021). Resistance exercise provides a strong mechanical stimulus that is classically associated with increases in muscle strength and protein synthesis and is often linked to favorable adaptations in muscle mass regulation (Geirsdottir et al., 2012; Ato et al., 2019). While both exercise forms are beneficial, it remains incompletely understood whether combining aerobic and resistance exercise produces additive or complementary effects on diabetic sarcopenia, particularly at the level of muscle protein regulation and intracellular signaling. Clarifying how different exercise modalities influence skeletal muscle adaptation under diabetic conditions therefore remains an important research objective.

In recent years, increasing attention has focused on myokines as potential mediators linking exercise stimuli to systemic and local tissue adaptations. Irisin, a peptide cleaved from fibronectin type III domain-containing protein 5, is released from skeletal muscle in response to exercise and has been implicated in the regulation of energy metabolism, insulin sensitivity, and muscle remodeling (Boström et al., 2012; Chen et al., 2016). Circulating irisin levels have been reported to correlate with muscle mass, muscle strength, and metabolic status, and reduced irisin concentrations have been observed in individuals with sarcopenia and T2DM (Chang et al., 2017; Oguz et al., 2021). Experimental studies further suggest that irisin may exert pro-myogenic or anti-atrophic effects under certain pathological conditions (Reza et al., 2017). Despite these findings, the downstream signaling mechanisms through which irisin may influence skeletal muscle adaptation remain incompletely defined. Although integrin αVβ5 has been identified as a functional receptor for irisin in bone and adipose tissue (Kim et al., 2018), skeletal muscle expresses high levels of integrin α7β1, a muscle-enriched integrin complex that plays a critical role in mechanotransduction, muscle integrity, and adaptive remodeling (Boppart et al., 2006). Whether exercise-associated changes in irisin are accompanied by coordinated modulation of integrin α7β1 expression and downstream anabolic signaling in diabetic skeletal muscle remains unclear.

Therefore, this study aimed to investigate the effects of aerobic exercise, resistance exercise, and combined aerobic–resistance exercise on skeletal muscle protein content, muscle mass–related phenotypes, and metabolic parameters in a rat model of T2DM. In addition to assessing morphological and biochemical indices of muscle adaptation, this study examined changes in irisin levels and the coordinated expression of integrin α7β1 and key components of the PI3K/AKT/mTOR signaling pathway. By comparing distinct exercise modalities under identical diabetic conditions, this work sought to provide insight into how exercise-associated myokine signaling aligns with skeletal muscle protein accretion and metabolic regulation in diabetes.

## 2 Methods

### 2.1 Experimental animals

Fifty male Sprague–Dawley rats (6 weeks old) were obtained and housed under controlled environmental conditions with a 12 h light–dark cycle, ambient temperature of 22 °C–24 °C, and free access to food and water. All animals underwent a 1 week acclimatization period prior to experimental procedures. All experimental protocols were approved by the Biomedical Research Ethics Committee of Hunan Normal University (Approval No. 2021345) and were conducted in accordance with institutional guidelines for the care and use of laboratory animals.

### 2.2 Induction of T2DM

After acclimatization, rats were randomly assigned to a normal control group (NC, n = 10) or a diabetes induction group (n = 40). Rats in the NC group were fed a standard laboratory diet, whereas rats in the diabetes induction group were fed a high-fat diet for 4 weeks. Following this period, rats were fasted overnight (∼12 h) and administered a single intraperitoneal injection of streptozotocin (STZ; 35 mg/kg body weight), freshly dissolved in citrate buffer. Rats in the NC group received an equivalent volume of citrate buffer.

Fasting blood glucose was measured from the tail vein on days 3 and 8 after STZ injection using a handheld glucose meter. Rats with fasting blood glucose ≥7.8 mmol/L on both occasions were considered successfully diabetic and were included in subsequent experiments.

### 2.3 Experimental grouping

Successfully modeled diabetic rats were randomly allocated into four groups: diabetes control (DC, n = 10), diabetes aerobic exercise (DA, n = 10), diabetes resistance exercise (DR, n = 10), and diabetes combined aerobic–resistance exercise (DAR, n = 10). Rats in the NC and DC groups did not undergo exercise intervention. All animals had *ad libitum* access to food and water throughout the experimental period. Body weight and fasting blood glucose were recorded weekly.

### 2.4 Exercise intervention

Exercise intervention was conducted using a motorized animal treadmill (ZH-PT, HuaiBei ZhengHua Biological Instrument Co., Ltd., China). Prior to formal training, rats in the DA, DR, and DAR groups completed a 3 day adaptation period at 10–15 m/min for 30 min/day to familiarize them with treadmill running.

For aerobic exercise, rats in the DA group ran at a speed of 17 m/min, corresponding to moderate-intensity aerobic exercise in rodents. For resistance exercise, rats in the DR group ran at 15 m/min while carrying progressively increased external loads attached to the tail. Load intensities were set at 10% of body weight during weeks 1–2, 30% during weeks 3–4, 50% during weeks 5–6, and 70% during weeks 7–8. Each resistance training session consisted of repeated running bouts separated by brief rest intervals. This protocol was designed to provide a load-dominant mechanical stimulus during locomotion and, consistent with rodent treadmill-based resistance models, necessarily retained an endurance component rather than representing an isolated resistance paradigm.

Rats in the DAR group alternated between aerobic exercise (Mondays, Wednesdays, Fridays) and resistance exercise (Tuesdays, Thursdays, Saturdays). Exercise intensity, duration, and progression were matched to those of the DA and DR groups. All exercise-trained rats performed 60 min of exercise per day, 6 days per week, for eight consecutive weeks. Rats in the NC and DC groups were handled similarly but did not perform exercise.

**FIGURE 1:**
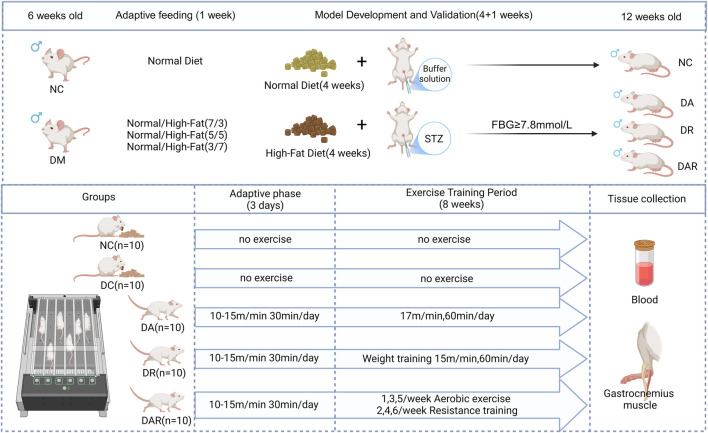
Experimental design and exercise intervention protocol.

A schematic overview of the experimental design, diabetes induction, group allocation, and exercise intervention timeline is presented in Figure 1.

### 2.5 Sample collection

At the end of the 8 week intervention period, rats were fasted overnight (∼12 h) and anesthetized by intraperitoneal injection of 1% sodium pentobarbital (40 mg/kg). Blood samples were collected from the thoracic aorta, allowed to clot at room temperature for 30 min, and centrifuged at 4 °C and 4,000 rpm. Serum was separated and stored at −20 °C until analysis.

Bilateral gastrocnemius muscles were excised, rinsed with cold saline, blotted dry, and weighed. The right gastrocnemius muscle was fixed in 4% paraformaldehyde for histological analysis, and the left gastrocnemius muscle was snap-frozen and stored at −80 °C for biochemical and protein analyses.

### 2.6 Biochemical measurements

Fasting blood glucose was measured using the glucose oxidase method with a commercial assay kit (Nanjing Jiancheng Bioengineering Institute, China). Serum insulin, glycated hemoglobin (HbA1c), and irisin levels in serum and muscle tissue were quantified using enzyme-linked immunosorbent assay kits (Shanghai Enzyme-Linked Technology Bio-Tech Co., Ltd., China) according to the manufacturers’ instructions. Insulin resistance was estimated using the homeostasis model assessment of insulin resistance (HOMA-IR), calculated as insulin (mIU/L) × fasting blood glucose (mmol/L)/22.5.

### 2.7 Histological analysis

Gastrocnemius muscles from three randomly selected rats per group were dehydrated, embedded in paraffin, and sectioned at 3 μm thickness. Sections were stained with hematoxylin–eosin (HE) to assess muscle fiber morphology and with Masson’s trichrome to evaluate collagen deposition and fibrosis. Images were captured using an Eclipse Ci-L light microscope (Nikon, Japan). Muscle fiber cross-sectional area was quantified using Image-Pro Plus 6.0 software. Collagen volume fraction was calculated from Masson-stained sections using standard image analysis procedures.

### 2.8 Skeletal muscle protein quantification

Total skeletal muscle protein concentration was determined using a bicinchoninic acid protein assay kit (Nanjing Jiancheng Bioengineering Institute, China) following homogenization of muscle tissue in lysis buffer.

### 2.9 Western blot analysis

Protein extraction from gastrocnemius muscle was performed using samples from six randomly selected rats per group. Tissues were homogenized in RIPA lysis buffer, and equal amounts of protein were separated by SDS–PAGE and transferred onto polyvinylidene fluoride (PVDF) membranes. Membranes were blocked with 5% non-fat milk at room temperature and incubated overnight at 4 °C with primary antibodies against MuRF1, Atrogin-1, PI3K, AKT, mTOR, integrin β1 (Abcam), and integrin α7 (Bio-Rad). After washing, membranes were incubated with horseradish peroxidase–conjugated secondary antibodies. Protein bands were visualized using enhanced chemiluminescence and quantified by densitometry using Image-Pro Plus 6.0 software. GAPDH or β-actin was used as the internal loading control, as appropriate.

### 2.10 Statistical analysis

Statistical analyses were structured around three biological questions: whether induction of diabetes produced a measurable pathological phenotype relative to normal controls (family 1: NC vs DC), whether exercise intervention rescued the diabetic phenotype relative to diabetic controls (family 2: DC vs DA, DR, DAR), and which exercise modality conferred greater efficacy among trained groups (family 3: DA vs DR, DAR; DR vs DAR). For each planned pairwise contrast, group differences were evaluated using two-sample tests (Welch’s *t*-test or Wilcoxon rank-sum test, as appropriate based on distributional characteristics). In addition, baseline-to-week-8, within-group changes for body weight were assessed using two-sample tests. *P* values were adjusted for multiple testing within each contrast family using the Holm method. Effect sizes were reported as Hedges’ *g* for parametric contrasts and Cliff’s *δ* for nonparametric contrasts. All tests were two-tailed, and *P* < 0.05 was considered statistically significant. Statistical analyses were performed using R. The data that support the conclusions of the study can be accessed at Figshare (DOI: 10.6084/m9.figshare.31286356).

## 3 Results

### 3.1 Metabolic phenotype

**FIGURE 2:**
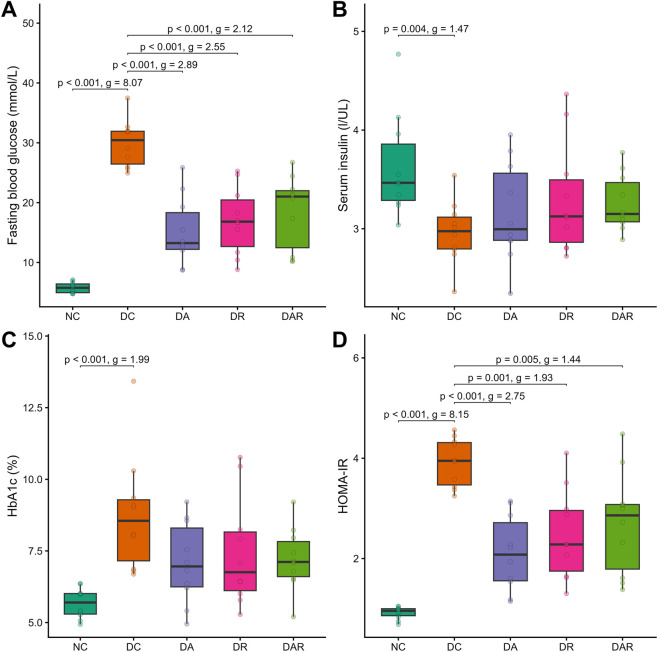
Effects of 8 week exercise on metabolic phenotype **(A–D)** Fasting blood glucose **(A)**, serum insulin **(B)**, glycated hemoglobin (HbA1c; **(C)** and insulin resistance index (HOMA-IR; **(D)** NC = normal control; DC = diabetic control; DA = diabetic aerobic exercise; DR = diabetic resistance exercise; DAR = diabetic combined aerobic–resistance exercise. Exact Holm-adjusted *P* values and effect sizes (Hedges’ *g* or Cliff’s δ, as appropriate) are shown.

Rats subjected to high-fat feeding followed by streptozotocin injection developed a stable diabetic phenotype. Compared with NC, DC exhibited significantly elevated fasting blood glucose, glycated hemoglobin, and HOMA-IR values, confirming successful induction of hyperglycemia and insulin resistance (Figure 2). Circulating insulin levels were also significantly lower in DC than in NC.

Exercise training improved selected metabolic parameters in diabetic rats. Compared with DC, fasting blood glucose and HOMA-IR were significantly reduced in DA, DR, and DAR (Figures 2A,D). In contrast, glycated hemoglobin levels did not differ significantly between DC and any exercise condition (Figure 2C). Circulating insulin levels were not significantly altered by exercise training, and no significant differences were observed among DA, DR, and DAR (Figure 2B).

No significant differences were detected among exercise modalities for fasting blood glucose, glycated hemoglobin, insulin, or HOMA-IR.

### 3.2 Body weight and muscle morphology

**FIGURE 3:**
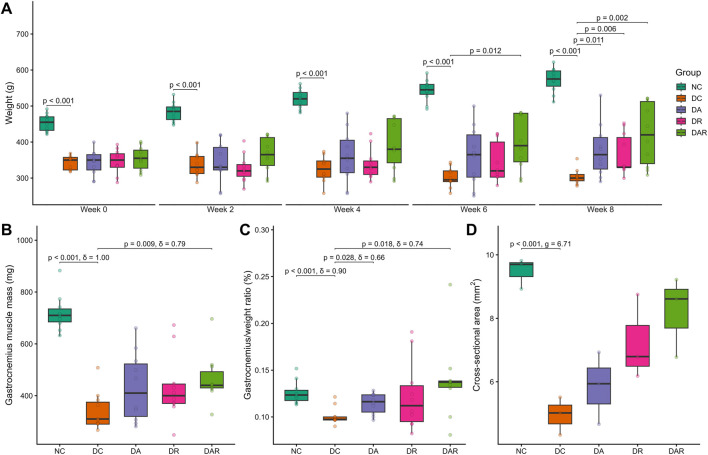
Effects of 8 week exercise on body weight and muscle morphology **(A)** Body weight changes **(B,C)** Gastrocnemius muscle mass **(B)** and gastrocnemius-to-body weight ratio **(C) (D)** Gastrocnemius muscle fiber cross-sectional area. NC = normal control; DC = diabetic control; DA = diabetic aerobic exercise; DR = diabetic resistance exercise; DAR = diabetic combined aerobic–resistance exercise. Exact Holm-adjusted *P* values and effect sizes (Hedges’ *g* or Cliff’s δ, as appropriate) are shown.

DC exhibited a progressive reduction in body weight over the experimental period compared with NC (Figure 3A). Within-group analysis showed that body weight increased significantly from week 0 to week 8 in NC (*P* = 0.026, Cliff’s *δ* = 1.00). In contrast, DC showed a reduction in body weight over the same period that did not reach statistical significance (*P* = 0.056, Cliff’s *δ* = 0.86). No significant within-group changes in body weight were detected in DA, DR, or DAR across the intervention period.

Despite the absence of significant within-group weight gain, exercise training significantly attenuated diabetes-associated weight loss (Figure 3A). At week 8, body weight was significantly higher in DA, DR, and DAR compared with DC. No significant differences in body weight were observed among DA, DR, and DAR at any time point.

Of note, food intake was not directly measured in the present study; therefore, differences in body weight should be interpreted as reflecting net energy balance rather than isolated differences in caloric intake. Accordingly, exercise-associated changes in body weight may arise from differences in energy expenditure, substrate utilization, or tissue partitioning.

Assessment of skeletal muscle mass revealed significant reductions in gastrocnemius muscle weight, gastrocnemius-to-body weight ratio, and muscle fiber cross-sectional area in DC compared with NC (Figures 3B–D). Exercise training significantly increased absolute gastrocnemius muscle mass in DAR relative to DC, whereas no significant increases were observed in DA or DR (Figure 3B). The gastrocnemius-to-body weight ratio was significantly higher in DA and DAR compared with DC, but not in DR (Figure 3C). Muscle fiber cross-sectional area did not differ significantly between DC and any exercise condition (Figure 3D).

No significant differences in muscle mass indices or fiber cross-sectional area were observed among DA, DR, and DAR.

### 3.3 Skeletal muscle fibrosis

**FIGURE 4:**
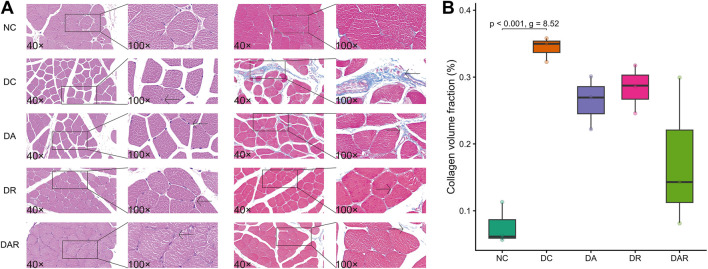
Effects of 8 week exercise on skeletal muscle histology and fibrosis **(A)** Representative hematoxylin–eosin and Masson’s trichrome–stained sections of gastrocnemius muscle from each group **(B)** Quantification of collagen volume fraction in skeletal muscle tissue. NC = normal control; DC = diabetic control; DA = DA; DR = diabetic resistance exercise; DAR = diabetic combined aerobic–resistance exercise. Exact Holm-adjusted *P* values and effect sizes (Hedges’ *g* or Cliff’s δ, as appropriate) are shown.

Histological analysis revealed uniform muscle fiber size and orderly arrangement in NC, whereas DC displayed greater fiber size heterogeneity and disrupted organization (Figure 4A). Exercise training did not result in statistically significant differences in these histological features relative to DC.

Masson’s trichrome staining showed increased interstitial collagen deposition in DC compared with NC (Figure 4A). Quantitative analysis confirmed a significantly higher collagen volume fraction in DC (Figure 4B). Exercise training did not significantly reduce collagen volume fraction in any exercise condition, and no significant differences were observed among DA, DR, and DAR.

### 3.4 Skeletal muscle protein content and atrophy signaling

**FIGURE 5:**
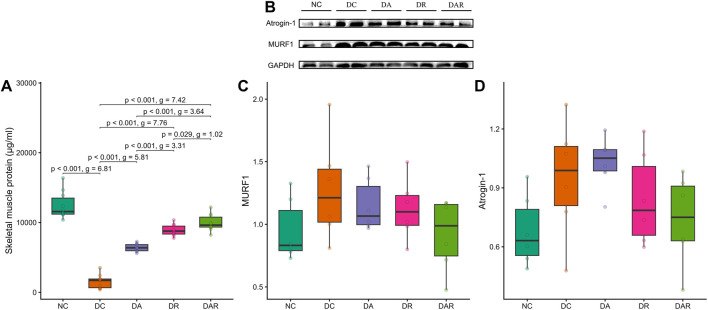
Effects of 8 week exercise on skeletal muscle protein content and atrophy signaling **(A)** Total skeletal muscle protein concentration **(B–D)** Representative Western blot images and quantitative analysis of muscle RING finger protein 1 (MuRF1) and atrogin-1 expression in gastrocnemius muscle. NC = normal control; DC = diabetic control; DA = diabetic aerobic exercise; DR = diabetic resistance exercise; DAR = diabetic combined aerobic–resistance exercise. Exact Holm-adjusted *P* values and effect sizes (Hedges’ *g* or Cliff’s δ, as appropriate) are shown.

Total skeletal muscle protein content was significantly reduced in DC compared with NC (Figure 5A). Exercise training significantly increased muscle protein content in DA, DR, and DAR relative to DC. Muscle protein levels were significantly higher in DR and DAR than in DA, and were also significantly higher in DAR than in DR.

Protein expression levels of the ubiquitin–proteasome markers MuRF1 and atrogin-1 did not differ significantly between NC and DC (Figures 5B–D). Exercise training did not significantly alter the expression of either marker relative to DC, and no significant differences were observed among DA, DR, and DAR.

### 3.5 Irisin–integrin–PI3K/AKT/mTOR signaling

**FIGURE 6:**
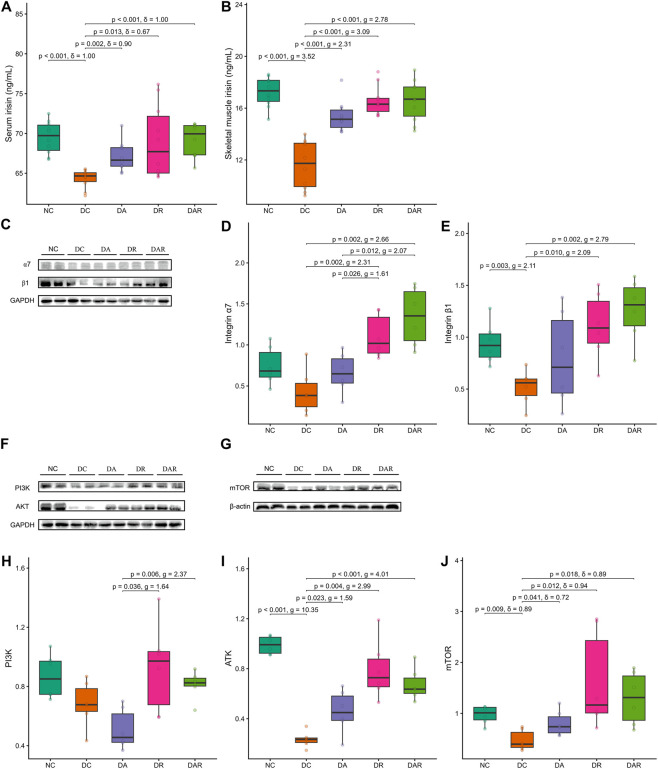
Associations between 8 week exercise and Irisin–integrin–PI3K/AKT/mTOR signaling **(A,B)** Serum irisin **(A)** and skeletal muscle irisin **(B) (C–E)** Representative Western blot images and quantitative analysis of integrin α7 and integrin β1 expression in gastrocnemius muscle **(F–J)** Representative Western blot images and quantitative analysis of phosphatidylinositol 3-kinase (PI3K), protein kinase B (AKT), and mammalian target of rapamycin (mTOR) expression. NC = normal control; DC = diabetic control; DA = diabetic aerobic exercise; DR = diabetic resistance exercise; DAR = diabetic combined aerobic–resistance exercise. Exact Holm-adjusted *P* values and effect sizes (Hedges’ *g* or Cliff’s δ, as appropriate) are shown.

Both serum and skeletal muscle irisin levels were significantly reduced in DC compared with NC (Figures 6A,B). Exercise training significantly increased serum irisin and skeletal muscle irisin levels in DA, DR, and DAR relative to DC. No significant differences in irisin levels were observed among DA, DR, and DAR.

Expression of integrin α7 did not differ significantly between NC and DC, whereas integrin β1 expression was significantly reduced in DC (Figures 6C–E). Exercise training significantly increased integrin α7 and integrin β1 expression in DR and DAR relative to DC, but not in DA. Integrin α7 expression was significantly higher in DR and DAR than in DA, whereas no significant difference was observed between DR and DAR.

PI3K expression did not differ significantly between NC and DC and was not significantly altered by exercise relative to DC (Figures 6F, H). However, PI3K expression was significantly higher in DR and DAR than in DA, with no significant difference between DR and DAR.

Expression levels of AKT and mTOR were significantly reduced in DC compared with NC (Figures 6F,G,I,J). Exercise training significantly increased AKT and mTOR expression in DA, DR, and DAR relative to DC. No significant differences were detected among DA, DR, and DAR for AKT or mTOR expression.

## 4 Discussion

This study examined how aerobic exercise, resistance exercise, and their combination influence skeletal muscle mass, protein content, and associated signaling pathways in a rat model of T2DM. The central finding is that exercise training induces a robust increase in skeletal muscle protein content accompanied by coordinated activation of anabolic signaling pathways, despite minimal structural remodeling of muscle fibers and no detectable suppression of canonical atrophy markers. Together, these results identify a non-hypertrophic anabolic adaptation as a dominant mode of skeletal muscle response to exercise under diabetic conditions.

### 4.1 Muscle-level adaptations: protein accretion without hypertrophy

Consistent with established models of T2DM, diabetic rats displayed pronounced metabolic dysfunction, including hyperglycemia and insulin resistance, alongside reductions in skeletal muscle mass and total muscle protein content. Exercise training improved selected metabolic indices across all exercise modalities; however, glycated hemoglobin and circulating insulin levels were not significantly altered, indicating that metabolic rescue was partial rather than complete. Importantly, these metabolic improvements were not accompanied by consistent increases in muscle fiber cross-sectional area or reductions in fibrosis, underscoring that metabolic and structural adaptations can be dissociated in diabetic skeletal muscle (Espino-Gonzalez et al., 2024).

A defining outcome of this study is that exercise-induced increases in muscle protein content occurred largely in the absence of overt hypertrophy. Absolute gastrocnemius muscle mass increased significantly only with combined aerobic–resistance exercise, whereas muscle fiber cross-sectional area remained unchanged across all exercise modalities. These findings demonstrate that protein accretion can occur independently of detectable fiber enlargement, supporting the existence of a non-hypertrophic anabolic state (Joanisse et al., 2013). Such an adaptation may reflect increased myofibrillar protein density, altered intracellular protein composition, or expansion of non-contractile protein pools rather than classical hypertrophic growth. This distinction is particularly relevant in diabetic muscle, where structural remodeling may be constrained by extracellular matrix accumulation, impaired satellite cell activity, or altered neuromuscular activation (Orlando et al., 2016; Han et al., 2022; Wu and Coletta, 2025).

### 4.2 Protein turnover: anabolic dominance without proteolytic suppression

Notably, exercise-induced protein accretion was not accompanied by suppression of canonical ubiquitin–proteasome system markers. Expression of MuRF1 and atrogin-1 did not differ significantly between normal and diabetic controls and was not altered by any exercise modality. These results indicate that diabetes-related muscle protein loss in this model is not characterized by sustained activation of these E3 ubiquitin ligases and that exercise-mediated increases in muscle protein content occur independently of detectable changes in these proteolytic pathways. This observation challenges the prevailing assumption that exercise preserves muscle mass in diabetes primarily through inhibition of ubiquitin–proteasome–mediated degradation and instead suggests that enhanced anabolic processes dominate muscle adaptation under these conditions (Bodine and Baehr, 2014; O’Neill et al., 2018).

### 4.3 Signaling patterning: coordinated but non-linear anabolic activation

At the molecular level, these tissue-level dissociations are paralleled by coordinated but non-uniform changes across the irisin–integrin–PI3K/AKT/mTOR signaling axis. Both serum and skeletal muscle irisin levels were reduced in diabetic rats and increased with exercise training, with skeletal muscle irisin exhibiting an exercise-general response and circulating irisin showing greater sensitivity to resistance-containing interventions. Downstream, AKT and mTOR expression increased across all exercise modalities, whereas integrin α7, integrin β1, and PI3K displayed modality-dependent changes, with greater engagement in resistance and combined exercise conditions. Importantly, PI3K expression was not reduced in diabetic muscle relative to controls, nor was it uniformly “rescued” by exercise, indicating that exercise-related differences in PI3K signaling reflect patterning among exercise modalities rather than normalization from a diseased baseline.

Collectively, these findings support a model in which exercise induces patterned activation of anabolic signaling networks rather than uniform engagement of a linear signaling cascade. While irisin has been proposed as a myokine linking muscle contraction to anabolic and metabolic adaptation (Boström et al., 2012; Guo et al., 2023), and integrin α7β1 is a key mediator of mechanotransduction in skeletal muscle (Boppart et al., 2006), the present data demonstrate coordinated associations rather than receptor-mediated causality. Accordingly, the results should be interpreted as evidence of signaling alignment within an exercise-responsive network, not as proof of a direct irisin–integrin–PI3K–mTOR pathway.

### 4.4 Modality specificity without global superiority

An important implication of the present findings is that exercise modality confers selective, endpoint-specific advantages rather than universal superiority across outcomes. Combined aerobic–resistance exercise produced the largest increases in muscle protein content and was associated with greater engagement of selected integrin- and PI3K-related signaling components, suggesting heightened sensitivity to mechanical loading–related cues. However, modality differences were not observed for metabolic indices, muscle fiber cross-sectional area, fibrosis, or core AKT/mTOR responses. These results indicate that exercise modality exerts domain-specific effects, reinforcing the need to evaluate exercise adaptations across multiple biological levels rather than inferring overall efficacy from isolated endpoints.

### 4.5 Neuromuscular context and translational boundaries

Interpretation of the present muscle-level adaptations requires consideration of the broader neuromuscular system and the inherent translational boundaries of the experimental model. In humans, resistance training–induced improvements in strength and muscle mass are strongly influenced by neural and integrative neuromuscular adaptations, particularly during early phases of training (Lecce et al., 2026a). Extensive physiological evidence demonstrates that increases in supraspinal drive, spinal excitability, motor unit recruitment and discharge behavior, and neuromuscular junction stability contribute substantially to exercise responsiveness and can precede, or occur independently of, overt muscle hypertrophy (Egan and Sharples, 2023; Furrer et al., 2023).

In parallel, T2DM-related sarcopenia in humans is increasingly recognized as a mixed myogenic and neurogenic condition. Beyond intrinsic alterations in muscle protein metabolism, diabetes is associated with impairments in neural activation, motor unit remodeling, excitation–contraction coupling, neuromuscular junction integrity, and extracellular matrix organization, all of which contribute to reduced muscle quality and diminished adaptive capacity (Lecce et al., 2026b). These neuromuscular deficits are considered major determinants of muscle weakness and functional decline in diabetic populations and may constrain both the magnitude and nature of exercise-induced adaptations.

By contrast, rodent treadmill- and tail-load–based exercise paradigms primarily emphasize peripheral mechanical loading and metabolic engagement of skeletal muscle and do not capture the full spectrum of supraspinal, cortical, or fine motor unit–level remodeling characteristic of human resistance training. In the present study, no direct indices of neural adaptation—such as motor unit number estimation, nerve conduction properties, neuromuscular junction morphology, or electromyographic recruitment patterns—were assessed. Consequently, the observed associations between exercise training, muscle protein accretion, and anabolic signaling should be interpreted as muscle-level contributors within a broader neuromuscular adaptive process, rather than as sufficient explanations for exercise-induced mitigation of diabetic sarcopenia.

Within this context, the present findings identify a reproducible pattern of exercise-responsive muscle adaptations—namely increased protein accretion and coordinated activation of anabolic signaling pathways—that may translate across species. At the same time, they underscore that recovery of neural, motor unit, and functional adaptations central to resistance training efficacy in humans cannot be inferred from muscle-intrinsic molecular outcomes alone. These considerations emphasize the importance of integrating molecular, neural, and functional endpoints in future translational studies and caution against overextension of muscle-level findings when extrapolating to human neuromuscular performance.

### 4.6 Translational application

The present findings have direct implications for exercise-based strategies aimed at mitigating diabetic sarcopenia. Notably, the results demonstrate that exercise can promote muscle protein accretion and anabolic signaling without inducing overt hypertrophy or reversing fibrosis. This pattern of adaptation may be particularly relevant for older adults with T2DM, individuals with early or moderate sarcopenia, or patients with limited tolerance for high-load resistance training, in whom hypertrophic responses are often blunted (Hansen et al., 2025).

Resistance-containing exercise modalities preferentially engaged integrin-associated and PI3K-related signaling, whereas exercise-general effects predominated for AKT/mTOR activation, suggesting that multimodal exercise programs may provide complementary benefits at the molecular level. Rather than prioritizing hypertrophy as the sole therapeutic goal, exercise prescriptions for diabetic populations may benefit from emphasizing protein accretion, signaling responsiveness, and metabolic support, particularly during early or intermediate stages of intervention.

With respect to exercise dose and feasibility, the rodent protocol used here represents a high-frequency, moderate-intensity stimulus relative to established rodent endurance and resistance paradigms, designed to ensure consistent mechanical and metabolic engagement. Although direct quantitative translation to humans is inappropriate, this paradigm is physiologically more analogous to regular moderate-intensity aerobic activity combined with low-to-moderate load resistance exercise performed on most days of the week. Such patterns align with current clinical recommendations for individuals with T2DM (Kanaley et al., 2022; American Diabetes Association Professional Practice Committee, 2024) and are feasible for many, though not all, patient populations. Importantly, diabetes-related neuromuscular impairments may limit tolerance to high-volume or high-load exercise in frail individuals, underscoring the need for progressive, individualized, and multimodal exercise prescriptions (Lecce et al., 2026b).

## 5 Conclusion

This study demonstrates that exercise training in diabetic skeletal muscle elicits a non-hypertrophic anabolic adaptation, characterized by increased muscle protein content and patterned activation of anabolic signaling pathways in the absence of overt fiber hypertrophy, fibrosis reversal, or suppression of canonical atrophy markers. While aerobic, resistance, and combined exercise modalities all promoted anabolic signaling and protein accretion, resistance-containing interventions preferentially engaged integrin- and PI3K-associated components, indicating domain-specific modality effects rather than global superiority.

By highlighting protein accretion and signaling patterning as central outcomes, these findings refine current understanding of skeletal muscle adaptation to exercise under diabetic conditions and provide a conceptual framework for designing translational strategies aimed at preserving muscle health and neuromuscular adaptability in T2DM.

## Data Availability

The datasets presented in this study can be found in online repositories. The names of the repository/repositories and accession number(s) can be found in the article/supplementary material.
